# Supplementary material for: Risk Factors for Recurrent Hip Fractures Following Surgical Treatment of Primary Osteoporotic Hip Fractures in Chinese Older Adults
Source: Diseases. 2025 Oct 27;13(11):351. doi: 10.3390/diseases13110351 (PMC12651263; doi:10.3390/diseases13110351)
Supplement: Supplementary file 1 [file diseases-13-00351-s001.zip › Supplementary Table S1.docx]

**Supplementary Table S1** Demographic information and clinical characteristics of mortality in patients following surgical treatment of primary osteoporotic hip fracture surgery

|  | Living  (n=310) | Death  (n=66) | *P* value |
| --- | --- | --- | --- |
| Gender, n (%) |  |  | 0.931 |
| female | 238(76.8) | 51(77.3) |  |
| male | 72(23.2) | 15(22.7) |  |
| Age, n (%) |  |  | 0.002 |
| 65-84 years | 201(64.8) | 29(43.9) |  |
| ≥85 years | 109(35.2) | 37(56.1) |  |
| BMI (kg·m^-2^), n (%) |  |  | 0.731 |
| ＜19 | 218(70.3) | 45(68.2) |  |
| ≥19 | 92(29.7) | 21(31.8) |  |
| ASA, n (%) |  |  | 0.089 |
| 1 | 85(27.4) | 27(40.9) |  |
| 2 | 204(65.8) | 36(54.5) |  |
| 3 | 21(6.8) | 3(4.5) |  |
| Site of primary hip fracture, n (%) |  |  | 0.259 |
| Left | 148(47.7) | 38(57.6) |  |
| Right | 162(52.3) | 28(42.4) |  |
| Surgical types of primary hip fracture, n (%) |  |  | 0.344 |
| Internal fixation | 126(40.6) | 31(47.0) |  |
| Hip arthroplasty | 184(59.4) | 35(53.0) |  |
| Types of primary hip fracture, n (%) |  |  | 0.471 |
| Femoral neck fracture | 216(69.7) | 43(65.2) |  |
| Intertrochanteric fracture, | 94(30.3) | 23(34.8) |  |
| Anesthesia, n (%) |  |  | 0.780 |
| General anesthesia | 249(80.3) | 54(81.8) |  |
| Spinal anesthesia | 61(19.7) | 12(18.2) |  |
| Smoke, n (%) | 30(9.7) | 7(10.6) | 0.819 |
| Alcohol, n (%) | 46(14.8) | 11(16.7) | 0.819 |
| Blood transfusion | 71(22.9) | 10(15.2) | 0.164 |
| Concomitant underlying diseases |  |  |  |
| Hypertension, n (%) | 136(43.9) | 27(40.9) | 0.659 |
| Diabetes, n (%) | 60(19.4) | 11(16.7) | 0.612 |
| Heart attack, n (%) | 55(17.7) | 9(13.6) | 0.420 |
| Stroke, n (%) | 33(10.6) | 2(3.0) | 0.053 |
| Heart failure, n (%) | 6(1.9) | 1(1.5) | 0.819 |
| COPD, n (%) | 36(11.6) | 12(18.2) | 0.146 |
| Anxiety/Depression, n (%) | 5(1.6) | 0(0.0) | 0.299 |
| Dementia, n (%) | 13(4.2) | 4(6.1) | 0.507 |
| PD, n (%) | 34(11.0) | 11(16.7) | 0.195 |
| Deep vein thrombosis, n (%) | 9(2.9) | 2(3.0) | 0.956 |
| Sleep disturbance, n (%) | 8(2.6) | 0(0.0) | 0.187 |
| Antiosteoporosis drugs, n (%) | 134(43.3) | 15(22.7) | 0.002 |
| Duration of antiosteoporosis drug therapy (month) *Median (IQR)* | 36.0(6.5) | 25.8(17.1) | ＜0.001 |
| Laboratory variables |  |  |  |
| Hb level at admission (g·L^-1^) *Mean**±SD* | 105.1±17.4 | 108.3±17.9 | 0.175 |
| Albumin (g·L^-1^) , n (%) |  |  | 0.877 |
| ≥35 | 89(29.2) | 19(30.2) |  |
| ＜35 | 216(70.8) | 44(69.8) |  |
| eGFR(ml·min^-1^·1.73^-2^) *Median (IQR)* | 76.5(26) | 74.5(26) | 0.223 |
| Serum calcium (mmol·L^-1^) *Mean±SD* | 2.16±0.14 | 2.13±0.14 | 0.154 |
| 25-hydroxyvitamin D (nmol·L^-1^) *Median (IQR)* | 32.5(21.1) | 28.0(27.8) | 0.865 |

**Abbreviations**: BMI, body mass index; ASA, American society of anesthesiologists; COPD, chronic obstructive pulmonary disease; PD, Parkinson’s disease; IQR, interquartile range; SD, standard deviation.
